# United States Medical School Academic Faculty Workforce Diversity, Institutional Characteristics, and Geographical Distributions From 2014-2018

**DOI:** 10.7759/cureus.22292

**Published:** 2022-02-16

**Authors:** Dotun Ogunyemi, C. Camille Okekpe, Devin R Barrientos, Tina Bui, Melanie N Au, Sangeeta Lamba

**Affiliations:** 1 Graduate Medical Education, Arrowhead Regional Medical Center, Colton, USA; 2 Women's Health, Arrowhead Regional Medical Center, Colton, USA; 3 Medical Education, California University of Science and Medicine, Colton, USA; 4 Obstetrics and Gynecology, Arrowhead Regional Medical Center, Colton, USA; 5 Diversity and Inclusion, New Jersey Medical School, Rutgers University, Newark, USA

**Keywords:** underrepresented in medicine, equity, gender diversity, workforce, medical school faculty

## Abstract

Purpose

Academic healthcare workforce diversity is important in addressing health disparities. Our goal was to evaluate trends and associations in faculty diversity of United States (US) medical schools over a five-year period.

Methods

We analyzed the Association of American Medical Colleges (AAMC) Faculty Roster data of 151 US medical schools from 2014-2018. Outcome faculty variables were female gender, underrepresented in medicine (UiM), age, and professorial representation. Predictor variables included geographical distributions, and institutional characteristics. Statistical analysis included Jonckheere-Terpstra test, ANOVA, and regression analysis.

Results

Female faculty increased from 37.6% to 40.4% (p<0.001), senior faculty (age >60 years) from 22.6% to 25.9% (p=0.001) while UiM faculty stayed relatively flat from 9.74% to 10.08% (p=0.773). UiM [adjusted odds ratio (aOR) = 0.39, p=0.015], and female faculty (aOR=0.3, p=0.001) had independently significantly decreased associations with professorial representation, while senior faculty had increased associations (aOR=3.82, p<0.001). Significant independent differences occurred in female, UiM, and professorial faculty distributions within US regions; Hispanic faculty were highest in Southwest (6.57%) and lowest in Midwest region (1.59%), while African-American faculty were highest in Southeast (8.15%) but lowest in the West (3.12%). UiM faculty had significantly independent decreased associations with MD/PhD degree (aOR=0.30, p=0.004) and higher US ranking institutions (aOR=0.45, p=0.009).

Conclusions

From 2014 to 2018, female faculty increased modestly while the UiM faculty trend remained flat. Female and UiM faculty were less represented at the professor level. UiM faculty were less represented in higher-ranking institutions. Geographic location is associated with faculty diversity.

## Introduction

The importance of diversity in the United States (US) healthcare workforce in reducing health disparities is well recognized [1-3]. The Diversity 3.0 initiative proposed in 2010 by the Association of American Medical Colleges (AAMC) emphasizes that diversity and inclusion in academic medicine are important factors for addressing health disparities in the US [4]. A diverse academic faculty serves as important role models for learners and facilitates the training of diverse physicians who are more likely to practice in underserved and disadvantaged communities [1]. Academic faculty diversity further promotes innovative biomedical, and community-based participatory research that can address disparities in health access and outcomes of marginalized communities [1]. Furthermore, physician diversity has been linked to better patient outcomes [2-3].

Faculty who are historically underrepresented in medicine (UiM) include those who self-identify their race/ethnicity to include Black or African-American, Hispanic, or Latino, Alaskan Native, American Indian, and Native Hawaiian or Other Pacific Island (OPI) populations [5]. As of 2016, faculty identifying as UiM constituted approximately 8% of all medical faculty positions demonstrating only a 1% increase in 20 years from 1993 [6]. UiM faculty have also been shown to have fewer publications and were less likely to be promoted and retained in academic careers [7].

As academic faculty mature and age, there may be concerns about career flexibility regarding health, varying family needs, burnout, and changes in personal expectations. Institutions must therefore consider careful succession planning and finances since replacing retiring academic faculty is costly. Recruitment, training, and cost of the learning curve can often total more than $200,000 per faculty. Furthermore, vacancies especially of experienced and highly qualified faculty in leadership positions may also be harder to fill [8].

For institutions striving for inclusive and academic excellence, programs supporting recruitment, promotion, and retention of a diverse academic faculty are required. To address equity gaps for faculty diversity and develop effective interventions, it is crucial to have current knowledge of factors influencing contemporary faculty diversity [9]. Studies show that women in science are under-cited, under-promoted, and professionally under-recognized relative to their male counterparts [10-11]. For example, Mader et al. noted that women and African-Americans are better represented in faculty and leadership positions at Historically Black Colleges and Universities (HBCU), and similarly women and Latino/Hispanic identifying faculty were better represented at medical schools in Puerto Rico [12]. Carr et al. also noted a paucity of gender equity-focused programs in academic medicine [13]. Therefore, identifying geographic, institutional, workplace, and learning environment factors that may impact faculty diversity is important. Analyzing current faculty diversity trends will assist in identifying and defining equity gaps and measuring any progress made over time which is an important first step in addressing disparities in health care. Therefore, the objective of this study was to utilize the cohort of United States Medical School faculty over a five-year period from 2014-2018 to assess trends of gender, age, and race/ethnic diversity of faculty. A second objective was to analyze the distribution of institutional and faculty diversity characteristics by United States geographic regions.

## Materials and methods

We analyzed data from the Association of American Medical Colleges (AAMC) Faculty Roster Benchmark Report which included information on the faculty of 151 U.S. medical schools from 2014-2018 [14]. Specific data extracted were: Age groups (<=29, 30-39, 40-49, 50-59, 60-69, 70+); race/ethnicity which included: Non-Hispanic White (original peoples of Europe, the Middle East, or North Africa), African-American (origins in any of the Black racial groups of Africa), Hispanic, Asian (original peoples of the Far East, Southeast Asia, or the Indian subcontinent), Native Hawaiian or Other Pacific Islander (original peoples of Hawaii, Guam, Samoa, or other Pacific Islands), American Indian/Native Alaskan (original peoples of North and South America); gender (male, female); faculty degrees (MD, MD-PhD, PhD); and academic ranks (Professor, Associate Professor, Assistant Professor, Instructor) [15].

From the 2019 US News and World Report Ranking database for medical schools, we extracted data on: medical school ranking scores in research and primary care, full-time faculty-student ratio, total number of students enrolled in the medical school, National Institute of Health (NIH) funds granted to medical school and affiliated hospitals (in millions), NIH research grant funds per faculty member (in thousands), graduates entering primary care specialties, median undergraduate grade point average (GPA), Medical College Admission Test (MCAT) total score, and acceptance rates [16]. From the websites of each medical school, we extracted the year the school was established and if the school is a public or private school. We also categorized the geographical locations of the medical schools into the five US regions of Midwest, Northeast, Southeast, Southwest, and West [17-18].

We measured four categorical outcome variables: female gender, UiM group (faculty identifying as African-American, Hispanic, Native Hawaiian or Other Pacific Islander (OPI), American Indian/Native Alaskan), senior faculty (defined as ≥60 years of age), and professorial representation. We analyzed the percentages of the four outcome variables in all the medical schools. The values of each outcome variable of interest were divided into four equal intervals to calculate the 25th, 50th, and 75th percentiles. Values that were above the 75th percentile were categorized as the uppermost percentile and the lower quartiles included values within the three lower quartiles. We used the uppermost quartile as the desired outcome variable and in our analyses, we compared medical schools within the uppermost quartile versus those with the lower three quartiles. The values were calculated as follows: For female faculty, the uppermost quartile included medical schools with the percentage of female faculty at 42.4% or higher, while female lower quartiles included medical schools with the percentage of female faculty less than 42.4%. For UiM faculty, the uppermost quartile included medical schools with the percentage of UiM faculty at 8.06% or higher, and UiM lower quartiles were medical schools with the percentage of UiM faculty less than 8.06%. For senior faculty, the uppermost quartile included medical schools with the percentage of senior faculty (>60 years of age) at 27% or higher and senior faculty lower quartiles were medical schools with the percentage faculty who were less than 60 years of age. Lastly, for professorial representation, the uppermost quartile included medical schools with the percentage of professors at 26.6% or higher and professorial representation lower quartiles included medical schools with the percentage of professors less than 26.6%.

Jonckheere-Terpstra test was performed to determine the significance of the yearly trends of UiM groups, senior faculty, and female gender over the five years of the study period. Jonckheere-Terpstra test is a rank-based nonparametric test that is used to determine if there is a statistically significant trend between an ordinal independent variable and a continuous or ordinal dependent variable.

A one-way analysis of variance (ANOVA) was used to determine statistically significant differences in the distributions of the outcome variables (female gender, UiM, senior faculty, and professorial representation) across institutional characteristics of the medical schools in the five U.S. regions of Midwest, Northeast, Southeast, Southwest, and West [17-18]. The institutional characteristics were measured using variables from the 2019 US News & World ranking listed above [16].

Logistic regression analysis was used to assess the independent associations of the female gender, UiM, senior faculty, and professorial representation. The logistic regression analysis determines independent significant associations of multiple variables with one dichotomous dependent variable and provides adjusted odds ratios. The beta coefficient is also reported which indicates that for every 1-unit increase in the predictor variable, the outcome variable will increase or decrease by the beta coefficient value, holding all other predictors constant. A p-value of <0.05 with two-tailed significance was taken as significant. IRB review was obtained and the study was categorized as exempt.

## Results

**Figure 1:**
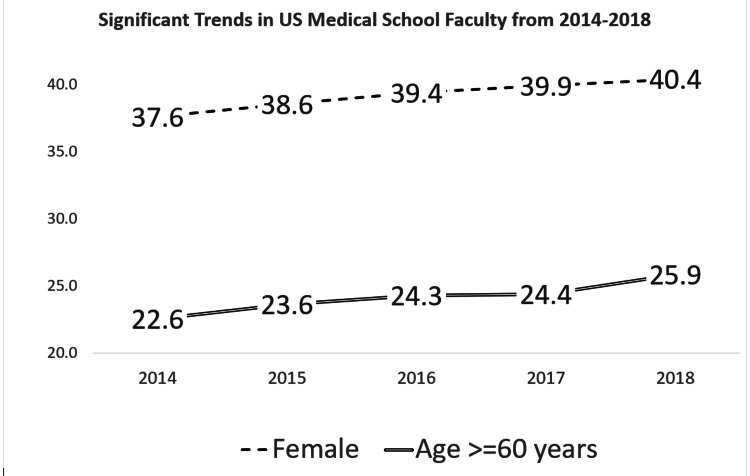
Statistically significant trends in US medical school faculty from 2014-2018 Female: Percentages of female faculty in United States medical schools from 2014-2018 Age >= 60 years: Percentage of faculty 60 years or older in United States medical schools from 2014-2018 Jonckheere-Terpstra test for both trends = p value <0.001 Underrepresented in medicine (UiM) trend was not statistically significant: 2014 = 9.74%; 2015 = 9.94%; 2016 = 9.91%; 2017 = 10.03% and 2018 = 10.08% (p=0.773) The Jonckheere-Terpstra test is a rank-based nonparametric test that is used to determine if there is a statistically significant trend between an ordinal independent variable and a continuous or ordinal dependent variable.

Over a five-year period, from 2014-2018, the trend for female faculty in US medical schools showed a statistically significant increase, from 37.6% to 40.4% (p <0.001). For overall UiM faculty there was no statistically significant change, from 9.74%-10.08% (p=0.773); with Hispanic from 5%-5.09%, American Indian/Native Alaskan from 0.91%-0.17%, Native Hawaiian/OPI from 0.09%-0.11%, and African-American from 4.47%-4.70%. Senior faculty (age >60 years) showed a statistically significant increase from 22.6% to 25.9% (p = 0.001) (Figure 1).

**Figure 2:**
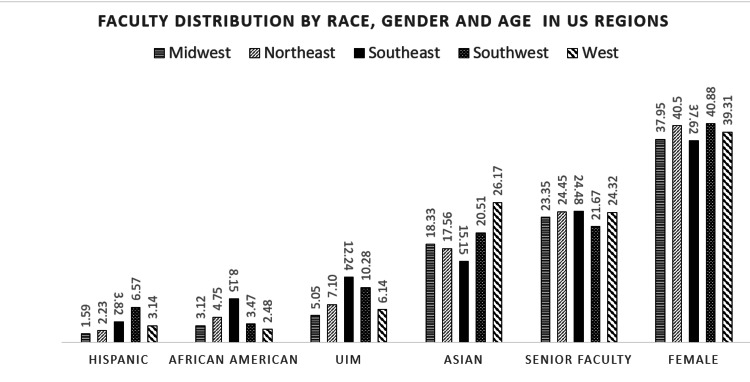
Faculty distribution by race/ethnicity, age, and gender and by location of medical schools in United States regions Values are from the Association of American Medical Colleges (AAMC) Faculty Roster Benchmark Report and are in percentages. * One-way analysis of variance (ANOVA) was used to determine statistically significant differences. All the distributions above were statistically significant with p<0.001, except for senior faculty with a p-value of 0.04. Underrepresented in medicine (UiM) groups were: African American, Hispanic, Native Hawaiian or Other Pacific Islander (OPI), American Indian/Native Alaskan. Senior faculty: faculty aged 60 years or more.

US regions differed significantly in faculty distribution characteristics. For faculty who identify as Hispanic, the highest percentage was in the Southwest (6.57%) and lowest in the Midwest region (1.59%). For African-American faculty, the highest percentage was in the Southeast (8.15%) and lowest in the West (3.12%); while the highest Asian faculty percentage was in the West (26.17%) and lowest in the Southeast (15.15%) (p<0.001). Senior faculty were present in the highest percentage in the Southeast (24.48%) and lowest in Southwest US regions (21.67%), while female faculty were present in the highest percentages in the Southwest (40.88%) and lowest in the Southeast (37.62%) (p<0.001) (Figure 2).

**Figure 3:**
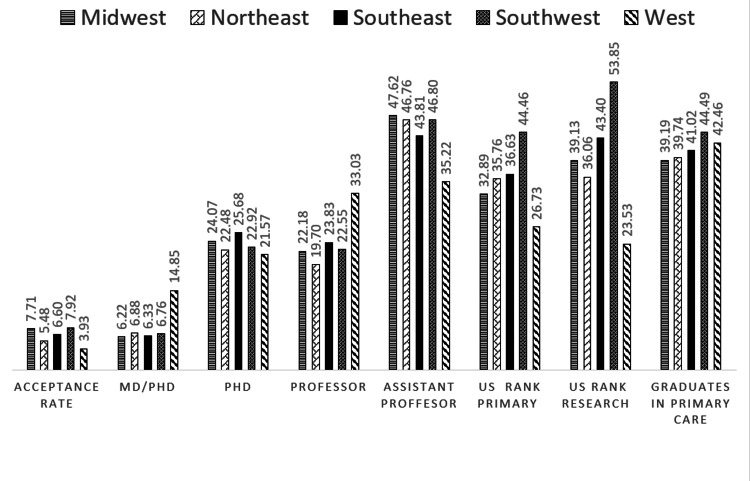
Distribution of academic institutional characteristics of medical schools by United States regions Values are from AAMC Faculty Roster Benchmark Report and are in percentages. * One-way analysis of variance (ANOVA) was used to determine statistically significant differences. All the distributions above were statistically significant with p<0.001, except for US rank primary p=0.029 Acceptance Rate: Percentage of applicants admitted to the medical school. US rank primary: US News & World Report ranking in primary care with 1 and a lower number being the most desirable. US rank research: US News & World Report ranking in research with 1 or a lower number being the most desirable. Grade point average (GPA): Median undergraduate GPA Medical College Admission Test (MCAT): Median MCAT total score

**Figure 4:**
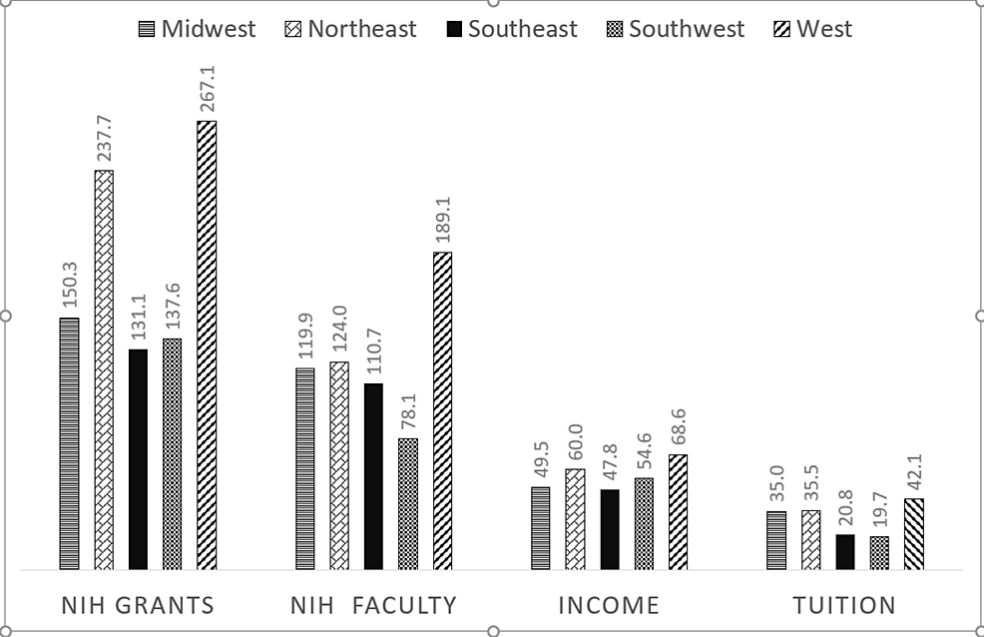
Distribution of research and institutional financial resources of medical schools by United States regions Values are from the Association of American Medical Colleges (AAMC) Faculty Roster Benchmark Report and are in percentages. * One-way analysis of variance (ANOVA) was used to determine statistically significant differences. All the distributions above were statistically significant with p<0.001. NIH grants: National Institute of Health (NIH) funds granted to medical school and affiliated hospitals (in millions) NIH faculty funds: NIH research grant funds per faculty member (in thousands) Income: Median income (in thousands) of the zip-code in which medical school is located Tuition: Tuition (in thousands) out of state charged by the medical school

For institutional characteristics, the highest admission MCAT scores, MD/PhD degrees, NIH grants, tuition, zip-code income, professor representation, and the lowest US News & Reports ranking in research and primary care and acceptance rates all occurred in the West US (P<0.001) (Figures 3, 4).

Medical schools in the uppermost quartile for female faculty (>42.4%) compared to medical schools with female faculty in lower quartiles, also had faculty who were significantly more likely to be UiM, have PhD degrees, and ranked as instructors (Table 1). For example, for instructor rankings the values were: [9.22% (standard error of the mean = 0.59) versus 4.08% (0.2), p<0.001], respectively. Medical schools in the upper quartile for female faculty were also located in zip codes with higher median incomes, with higher faculty-student ratio, and had more desirable (lower numbers) US ranking in research and primary care. For example, for US ranking in primary care the values were: [31.83 (2.45) versus 35.56 (2.47), p=0.02] (Table 1).

**Table 1:** Comparisons of sociodemographic and institutional characteristics of medical schools with higher percentages of female, senior or UiM or professor faculty members versus those with lower faculty percentages (significant findings) Table 1 includes 4 univariate analyses in successive sections in which medical schools were analyzed in binary groups by comparing medical schools in the uppermost quartile of each of the 4 variables of the female gender, UiM, senior faculty, and professors to the medical schools in the lower quartiles for each of the 4 listed variables. Values from AAMC Faculty Roster Benchmark Report (age, race, gender, degrees, ranks) are in percentages. All other values from US News & World Report Ranking database are means or medians. (): Standard error of the mean One-way analysis of variance (ANOVA) was used to determine statistically significant differences. UiM: Underrepresented in medicine groups included African American, Hispanic, Native Hawaiian or Other Pacific Islander (OPI), American Indian/Native Alaskan Female lower and uppermost quartiles: Medical schools with the percentage of female faculty less than 42.4% or greater than/equal to 42.4%, respectively UiM lower and uppermost quartiles: Medical schools with the percentage of UiM faculty less than 8.06% or greater than/equal to 8.06%, respectively Senior Faculty lower and uppermost quartiles: Medical schools with percentage faculty >60 years of age less than 27% or greater than/equal to 27%, respectively Professorial representation lower and uppermost quartiles: Medical schools with percentage of professors less than 26.6% or greater than/equal to 26.6%, respectively Income: Median income of the zip code in which medical school is located NIH faculty grant: NIH research grant funds per faculty member (in thousands) NIH grants: NIH funds granted to medical school and affiliated hospitals (in millions) Acceptance Rate: Percentage of applicants admitted to the medical school US ranking in primary care: US News & World Report ranking in primary care with 1 and a lower number being the most desirable US ranking in research: US News & World Report ranking in research with 1 and a lower number being the most desirable

Variables	Medical schools with female faculty in lower quartiles (n=386)	Medical schools with female faculty in uppermost quartile (n=134)	P-value
UiM	9.24 (0.58)	12.05 (1.42)	0.03
MD	66.28 (0.55)	59.02 (1.26)	<0.001
PhD	22.63 (0.44)	25.24 (0.94)	0.01
Assistant Professor	46.28 (0.53)	42.165 (0.82)	<0.001
Instructors	4.08 (0.2)	9.22 (0.69)	<0.001
NIH grant	165.423 (9.45)	258.486 (27.06)	<0.001
Income	52359.77 (903)	60516.95 (1698.48)	<0.001
US ranking Primary care	35.53 (1.45)	31.83 (2.45)	0.02
US ranking Research	38.95 (1.45)	35.56 (2.47)	0.04
Faculty student ratio	2.00 (0.07)	2.79 (0.21)	<0.001
Tuition out of state	29491.04 (1255.64)	35360.55 (2132.12)	0.019
Variables	Medical schools with UiM faculty in lower quartiles N=417	Medical schools with UiM faculty in uppermost quartile N=103	
Age > 60 years	23.17 (0.26)	27.26 (0.72)	<0.001
Female	38.77 (0.24)	40.52 (0.5)	<0.001
Male	60.65 (0.25)	59.26 (0.5)	0.007
MD/PhD	7.82 (0.3)	5.57 (0.28)	<0.001
Undergraduate GPA	3.78 (0.01)	3.73 (0.01)	<0.001
MCAT total score	512.97 (0.22)	510.45 (0.37)	<0.001
NIH faculty grant	133.84 (5.05)	79.17 (6.45)	<0.001
NIH grant	209.131 (11.88)	107.974 (12.46)	<0.001
Income	56127.83 (978.72)	49027.8 (1278.68)	<0.001
Tuition in state	17272.92 (736.52)	11715.83 (866.06)	<0.001
Tuition out of state	35452.07 (1258.87)	17412.45 (1807.71)	<0.001
US ranking Research	37.86 (1.39)	38.89 (2.79)	<0.001
Faculty student ratio	2.39 (0.09)	1.47 (0.1)	<0.001
Year school opened	1909.33 (2.67)	1942.79 (4.42)	<0.001
Variables	Medical schools with senior faculty in lower quartiles N=416	Medical schools with senior faculty in uppermost quartile N= 104	
UiM	7.09 (0.34)	17.50 (1.74)	<0.001
MD/PhD	6.96 (0.18)	8.04 (0.73)	0.04
MD	66.52 (0.48)	59.04 (1.38)	<0.001
PhD	21.49 (0.33)	28.012 (1.14)	<0.001
Professor	21.68 (0.35)	25.59 (0.90)	<0.001
Assistant Professor	46.09 (0.5)	43.03 (0.96)	0.002
Undergraduate GPA	3.78 (0.01)	3.69 (0.01)	<0.001
MCAT total score	513 (0.22)	510.38 (0.42)	<0.001
NIH faculty grant	130.29 (4.98)	93.90 (8.22)	<0.001
NIH grant	214.842 (11.86)	86.041 (11.13)	<0.001
Tuition in state	13262.86 (730.92)	7392.22 (970.03)	<0.001
Tuition out of state	32730.30 (1256.59)	26267.39 (2115.7)	0.008
Faculty student ratio	2.45 (0.09)	1.25 (0.1)	<0.001
Year school opened	1911.37 (2.77)	1934.24 (4.22)	<0.001
Variables	Medical schools with Professors in lower quartiles N=395	Medical schools with Professors in uppermost quartile N=125	
Age >60 years	23.14 (0.25)	27.47 (0.72)	<0.001
Female	39.87 (0.23)	37.43 (0.47)	<0.001
Male	59.92 (0.23)	61.8 (0.45)	<0.001
MD/PhD	6.29 (0.21)	10.20 (0.67)	<0.001
PhD	21.142 (0.36)	29.82 (1.04)	<0.001
Undergraduate GPA	3.76 (0.01)	3.781 (0.01)	0.025
Acceptance rate	6.53 (0.16)	5.63 (0.26)	0.005
NIH faculty grant	109.1 (4.76)	167.0 (9.02)	<0.001
Tuition in state	10922.03 (692.63)	13909.73 (1201.53)	0.031
US ranking Research	40.37 (1.45)	31.43 (2.41)	0.002
Year school opened	1914.15 (2.78)	1927.46 (4.24)	0.014

Medical schools in the uppermost quartile for UiM faculty (>8.06%) compared to other quartiles, had faculty that were significantly more likely to be female, senior, and less likely to have MD/PhD degrees. For example, the values for MD/PhD degrees were: [5.57% (0.28) versus 7.82% (0.3), p<0.001], respectively. Medical schools in the upper quartile for UiM faculty were significantly more likely to be located in zip codes with lower median incomes and have fewer NIH grants, lower MCAT and GPA admission scores, lower tuition rates, lower faculty-student ratios and were relatively newer schools with less desirable US rankings in research. For example, the value for NIH grants reported in million dollars was [79.17 (6.45) versus 133.84 (5.05), p-value <0.001], respectively (Table 1).

For medical schools with senior (>60 years of age) faculty in the uppermost quartile (>27%), the faculty were significantly more likely to be UiM, have MD/PhD or PhD degrees, and achieve professor ranking but were less likely to have MD degrees. For example, for UiM faculty the values were: [17.50% (1.74) versus 7.09% (0.34), p<0.001] respectively. Medical schools in the upper quartile for senior faculty had significantly fewer NIH grants, lower MCAT and GPA admission scores, with smaller faculty-student ratios, and were relatively newer schools with lower tuition rates. For example, for NIH grants reported in million dollars, values were: [93.90 (8.22) versus 130.29 (4.98), p <0.001], respectively (Table 1).

Medical schools ranked in the uppermost quartile (>26.64%) for professors, as compared to lower quartiles, were significantly more likely to have faculty with MD/PhD or PhD degrees, senior (>60 years old), and male faculty. For example, the values for MD/PhD were: [10.20% (0.67) versus 6.29% (0.21), p <0.001], respectively. These medical schools in the uppermost quartile for professors, secured significantly more NIH grants, had higher GPA admission scores, had lower acceptance rates but higher tuition rates, and more desirable US ranking in research. For example, the values for NIH grants reported in million dollars were, [167.0 (9.02) versus 109.1 (4.76)], respectively (Table 1).

Table 2 shows the results of the logistic regression analyses to determine significant independent associations of UiM groups, senior faculty, female gender. UiM faculty had statistically significant, independent associations with lower professor representation, fewer MD/PhD degrees, and less desirable US ranking in primary care. Senior faculty had statistically significant, independent associations with higher professor representation, but with less desirable US rankings in primary care and lower GPA scores. Female faculty had statistically significant independent associations with lower professorial representations, with medical schools located in higher-income zip-codes who attained more desirable US ranking in research, and lower GPA scores.

**Table 2:** Independent associations of sociodemographic and institutional characteristics of medical schools with higher proportions of female faculty, senior faculty, and underrepresented in medicine racial group faculty from 2014-2018 by logistic regression analysis (significant results) B: Coefficient estimate is the amount of increase (or decrease, if the sign of the coefficient is negative) in the predicted log odds of the dependent variable that would be predicted by a 1-unit increase (or decrease) in the predictor, holding all other predictors constant. Female gender, senior faculty (age >60 years), UiM (underrepresented in medicine groups) were the dependent variables analyzed in association with the independent variables list below as indicated for model fit. Variables analyzed for models included: United States regions, US ranking in primary care, US ranking in research, the income of zip-code, year of the start of medical school, median undergraduate grade point average (GPA), acceptance rates into medical school, graduates entering primary care, faculty-student ratio, NIH faculty grant, MD/PhD, professorial representation, female faculty, UiM faculty, Senior faculty. Only significant results are reported. US Regions*: regions in this categorization were West, Southeast, Southwest, Midwest regions, and Northeast United States regions. Acceptance Rate: Percentage of applicants admitted to the medical school. US ranking in primary care: US News & World Report ranking in primary care with 1 or a lower number being the most desirable. For this analysis, medical schools that ranked from number 1-20 (best desirable scores) were categorized as 2 and those that ranked greater than 20th position (less desirable scores) were categorized as 1. US ranking in research: US News & World Report ranking in research with 1 or a lower number being the most desirable. For this analysis, medical schools that ranked from number 1-20 (best desirable scores) were categorized as 2 and those that ranked greater than 20th position (less desirable scores) were categorized as 1.

Model with Underrepresented in Medicine (UiM) as the dependent variable				
	B	P-value	Odds Ratio	95% CI
Professorial rank	-0.938	0.015	0.39	0.18-0.84
US Region*	0.802	<0.001	2.23	1.74-2.85
US ranking in primary care	-0.779	0.009	0.46	0.26-0.83
MD/PhD	-1.198	0.004	0.30	0.14-0.68
Model with Female gender as the dependent variable				
	B	P-value	Odds Ratio	95% CI
Professorial rank	-1.247	0.001	0.29	0.14-0.61
US regions*	-.323	0.014	0.72	0.56-0.94
US ranking in research	0.973	0.016	2.65	1.2-5.85
Undergraduate GPA	-.323	0.014	0.72	0.56-0.94
Income	1.114	<0.001	3.05	1.87-4.95
Model with Seniors (<60 years of age) as the dependent variable				
	B	P-value	Odds Ratio	95% CI
Undergraduate GPA	-1.143	<0.001	0.32	0.22-0.47
US ranking in primary care	-2.293	<0.001	0.10	0.04-0.24
Professorial rank	1.339	<0.001	3.82	1.8-8.01

On regression analysis, faculty ranked as professors had statistically significant, decreased associations with females or UiM but increased associations with MD/PhD degrees. Faculty ranked as professors had independent, statistically significant associations with medical schools with more desirable US ranking in both research and primary care, lower faculty-student ratios, higher admission GPA scores, and more NIH grants (Table 3).

**Table 3:** Independent associations of sociodemographic and institutional characteristics with medical schools with higher proportions of professors from 2014-2018 by logistic regression analysis (significant results) B: Coefficient estimate is the amount of increase (or decrease, if the sign of the coefficient is negative) in the predicted log odds of the dependent variable that would be predicted by a 1-unit increase (or decrease) in the predictor, holding all other predictors constant. US Regions*: regions in this categorization were West, Southeast, Southwest, Midwest regions, and Northeast regions in the United States. Variables entered into the logistic regression models included: United States regions, US ranking in primary care, the income of zip-code, US ranking in research, year of the start of medical school, median undergraduate grade point average (GPA), median Medical College Admission Test (MCAT) total score, acceptance rates into medical school, graduates entering primary care, faculty-student ratio, MD/PhD degrees, and NIH faculty grant. Acceptance Rate: Percentage of applicants admitted to the medical school. US ranking in primary care: US News & World Report ranking in primary care with 1 or a lower number being the most desirable. For this analysis, medical schools that ranked from number 1-20 (best desirable scores) were categorized as 2 and those that ranked greater than 20th position (less desirable scores) were categorized as 1. US ranking in research: US News & World Report ranking in research with 1 or a lower number being the most desirable. For this analysis, medical schools that ranked from number 1-20 (best desirable scores) were categorized as 2 and those that ranked greater than 20th position (less desirable scores) were categorized as 1. National Institute of Health (NIH) faculty grant: NIH research grant funds per faculty member (in thousands) For seniors, variables entered were: United States regions, US ranking in primary care, US ranking in research, year medical school opened, median undergraduate GPA, acceptance rates into medical school, graduates entering primary care, faculty-student ratio. Female, UiM, professors, MD/PhD, NIH grant.

Model with Professorial rank as the dependent variable				
	B	P-value	Odds Ratio	95% C.I.

Undergraduate GPA	0.528	<0.001	1.7	1.24 – 2.32
Female	-1.065	0.004	0.35	0.17 - 0.71
US regions*	0.800	<0.001	2.23	1.78 - 2.79
US ranking in research	1.702	<0.001	5.48	2.16 - 13.96
US ranking in primary care	1.253	0.001	3.50	1.63 - 7.51
Faculty student ratio	-0.841	<0.001	0.43	0.30 - 0.62
UiM	-1.504	<0.001	0.22	0.10 - 0.49
MD/PhD degree	2.188	<0.001	8.92	4.61 - 17.26
NIH faculty grant	1.227	0.009	3.41	1.36 - 8.56

## Discussion

A finding of this study is that the percentage of female medical school faculty increased significantly from 2014 to 2018. This increase from approximately 38% to 41% still suggests inadequate representation in comparison to the general population since the US Census population estimated that 58.2% of the population were females from 2014-2018 [19]. Increased awareness of gender equity issues combined with program support such as the AAMC Group on Women in Medicine and Sciences (GWIMS) may have contributed to the recent increases in female faculty. However, our findings verifying that women were still significantly less likely to have representation at the professorial rank, confirms previous studies and ongoing gender inequity [13,20]. This inadequate representation of women in academic medicine may be described as a “leaky pipeline” which may be attributable to adverse institutional climate such as gender bias, harassment, disproportionate burden of service loads, and work-life imbalance such as childcare, eldercare, and home schooling demands on women’s career progression. Interventions that address the root cause of gender equity such as implicit bias training, inclusive search and recruitment processes, equitable leave policies, supportive mentoring, and effective institution-wide policies addressing harassment offer the most opportunity to repair the pathway [21]. Targeted, often affinity group-based, programs such as ELAM (Executive Leadership in Academic Medicine) may be useful to address the senior rank and leadership-related gender equity gaps.

Another finding from our study was a modest, statistically significant increase in the trend of faculty aged 60 years and older in US medical schools. This trend with the aging of the academic faculty workforce is expected to continue [22]. There are clear implications of this trend for institutions as they will need to engage in thoughtful and intentional succession planning.

Our results also showed that the UiM faculty trend was flat over the five-year period studied. The US population for the same time period reveals that US UiM-related population was 33.2%, thus the faculty proportion of 9.4% in our study highlights the vast UiM equity gap that remains in academic medicine. Our findings confirm data from prior studies over the last 30 years that demonstrate that UiM faculty growth is still stagnant [23-24]. This large equity gap is a likely ongoing reflection of the underlying structural racism and social injustice issues in the United States [25]. Another barrier is the lack of a critical mass since the absence of enough UiM faculty to create a community can produce a fear of isolation for UiM faculty [7].

Our results also showed that UiM faculty had a very low professor representation which is in support of previous studies [26-27]. Intervening early to strengthen the minority medical student pathways may help to improve faculty diversity [27]. A foundational strategy is for medical schools to partner with their communities in providing educational, and mentoring opportunities for disadvantaged and underrepresented students to improve the likelihood of medical school attainment [25]. There is strong evidence that faculty development programs and mentoring programs can increase retention, productivity, and promotion for UiM faculty in academic medicine [9]. Career development interventions and research mentoring and debt reduction programs are posited to increase academic medicine diverse faculty retention and promotion [9].

Our study further showed the influence of geographic regions on faculty diversity. UiM faculty were more likely to serve marginalized and disadvantaged communities as we noted that schools with the highest UiM faculty were more likely to be located in zip codes with lower median incomes. This has important implications as we seek ways to address regional health disparities. In general, UiM faculty percentages mirrored the minority population percentages of the US regions [27]. These trends suggest that faculty seek a sense of belonging not only within the academic center but also desire a community in which their families will thrive and be nurtured.

We believe our study is one of the few to investigate the association between the US News & Report-based ranking of medical schools and faculty diversity. Desirable rankings including NIH grant funding were independently associated with professorial representation. “Research-intensive schools” receiving the largest NIH grants would generate more publications leading to more career advancement [24]. However, there is a downside. Choi et al. showed that the 25 most research-intensive medical schools had significantly lower proportions of graduates practicing primary care compared to the other 96 less research-intensive medical schools [28]. They concluded that to meet the health care needs of the US population, research-intensive private medical schools should play a more active role in promoting primary care careers for their students and graduates.

Limitations

The data we utilized is self-reported, cross-sectional, and retrospectively collected with potential concerns regarding inaccuracies and incomplete data. Hence causal inferences cannot be inferred based on this study. The data includes a binary consideration of gender identity and may not be representative of all gender identities. Heterogeneity, variability, and subjectivity in criteria used for faculty promotion and professorial ranking across institutions make the generalizability of this data challenging. Factors that contribute to faculty diversity such as faculty resilience, burnout, and institutional climate were not assessed. The strength of our study is the comprehensive use of geographic data and institutional characteristics. In addition to gender and UiM specific faculty data, which are usually the focus of faculty diversity studies, we also investigated older age as a variable.

## Conclusions

Five-year trends for female and senior (>60 years) faculty showed statistically significant increases from 2014-2018 but remained flat for UiM faculty. Institutional and faculty diversity characteristics differ significantly in various regions of the United States. Female and UiM faculty are still significantly lagging in representation in the professorial ranks and UiM faculty are less likely to be represented in high-ranking medical institutions. Innovative measures such as holistic interviews, community-based pathway programs at all career levels, career development and mentoring programs, unconscious bias training, effective and equitable institution-wide policies, and salaries may be helpful in achieving gender and racial equity in academic medical faculty. More effort is required to improve the diversification of NIH grantors, more UiM faculty especially those in low-ranking institutions should be included in study sections with a focus on funding studies on social justice and disparities. To address regional racial equity and faculty diversity gaps, medical schools may consider developing robust relationships with diverse communities and implement innovative programs that increase integration and reduce isolation for UiM faculty.
